# “Rogue” neutrophil-subset [DEspR+CD11b+/CD66b+] immunotype is an actionable therapeutic target for neutrophilic inflammation-mediated tissue injury – *studies in human, macaque and rat LPS-inflammation models*

**DOI:** 10.3389/fimmu.2022.1008390

**Published:** 2022-10-04

**Authors:** Saskia Carstensen, Meike Müller, Glaiza L. A. Tan, Khristine Amber Pasion, Jens M. Hohlfeld, Victoria L. M. Herrera, Nelson Ruiz-Opazo

**Affiliations:** ^1^ Fraunhofer Institute for Toxicology and Experimental Medicine ITEM, Department of Biomarker Analysis and Development, Hannover, Germany; ^2^ Whitaker Cardiovascular Institute and Department of Medicine, Boston University School of Medicine, Boston MA, United States; ^3^ Department of Respiratory Medicine, Hannover Medical School, Hannover, Germany; ^4^ Member of the German Center for Lung Research (DZL), Hannover, Germany

**Keywords:** neutrophil subset, DEspR, LPS-acute inflammation tissue injury models, acute lung injury, LPS-brain encephalopathy, segmental LPS challenge

## Abstract

**Background and objective:**

The correlation (Rs > 0.7) of neutrophils expressing the dual endothelin1/signal peptide receptor (DEspR+CD11b+/CD66b+) with severity of hypoxemia (SF-ratio) and multi-organ failure (SOFA-score) in patients with acute respiratory distress syndrome (ARDS) suggest the hypothesis that the DEspR+ neutrophil-subset is an actionable therapeutic target in ARDS. To test this hypothesis, we conducted *in vivo* studies to validate DEspR+ neutrophil-subset as therapeutic target and test efficacy of DEspR-inhibition in acute neutrophilic hyperinflammation models.

**Methods:**

We performed tests in lipopolysaccharide (LPS)-induced acute neutrophilic inflammation in three species – human, rhesus macaque, rat – with increasing dose-dependent severity. We measured DEspR+CD66b+ neutrophils in bronchoalveolar lavage fluid (BALF) in healthy volunteers (HVs) 24-hours after segmental LPS-challenge by ChipCytometry, and DEspR+CD11b+ neutrophils in whole blood and BALF in an LPS-induced transient acute lung injury (ALI) model in macaques. We determined anti-DEspR antibody efficacy *in vivo* in LPS-ALI macaque model and in high-mortality LPS-induced encephalopathy in hypertensive rats.

**Results:**

ChipCytometry detected increased BALF total neutrophil and DEspR+CD66b+ neutrophil counts after segmental LPS-challenge compared to baseline (*P* =0.034), as well as increased peripheral neutrophil counts and neutrophil-lymphocyte ratio (NLR) compared to pre-LPS level (*P <*0.05). In the LPS-ALI macaque model, flow cytometry detected increased DEspR+ and DEspR[-] neutrophils in BALF, which was associated with moderate-severe hypoxemia. After determining pharmacokinetics of single-dose anti-DEspR[hu6g8] antibody, one-time pre-LPS anti-DEspR treatment reduced hypoxemia (*P* =0.03) and neutrophil influx into BALF *(P* =0.0001) in LPS-ALI *vs* vehicle mock-treated LPS-ALI macaques. *Ex vivo* live cell imaging of macaque neutrophils detected greater “intrinsic adhesion to hard-surface” in DEspR+ *vs* DEspR[-] neutrophils (*P <*0.001). Anti-DEspR[hu6g8] antibody abrogated intrinsic high adhesion in DEspR+ neutrophils, but not in DEspR[-] neutrophils (*P <*0.001). In the LPS-encephalopathy rat model, anti-DEspR[10a3] antibody treatment increased median survival (*P* =0.0007) and exhibited brain target engagement and bioeffects.

**Conclusion:**

Detection of increased DEspR+ neutrophil-subset in human BALF after segmental LPS-challenge supports the correlation of circulating DEspR+ neutrophil counts with severity measure (SOFA-score) in ARDS. Efficacy and safety of targeted inhibition of DEspR+CD11b+ neutrophil-subset in LPS-induced transient-ALI and high-mortality encephalopathy models identify a potential therapeutic target for neutrophil-mediated secondary tissue injury.

## Introduction

Neutrophil-mediated secondary tissue injury with feed-forward progression underlies multi-organ failure (MOF) in acute respiratory distress syndrome (ARDS) (1). To date, there is still no FDA-approved pharmacotherapy able to reduce mortality and severity (2). Clinical trial failures of therapies for ARDS (3) show the challenges in ARDS and shortfalls of therapeutic pathways tested that promoted: 1] endothelial integrity and efferocytosis (rosuvastatin (4), simvastatin (5), or adenosine induction by recombinant interleukin-beta1a or FP1201) (6), 2] bronchodilation by β-agonist salbutamol (7), 3] pulmonary vasodilation by nitric oxide (8), 4] anti-inflammation and efferocytosis by dexamethasone (9, 10), or cyclooxygenase-prostaglandin inhibition by aspirin, 5] epithelial integrity by keratinocyte growth factor (11), 6] neutrophil elastase blockade (12), and 7] tissue repair *via* mesenchymal stem cell therapies (13). Collectively, these clinical trial failures demonstrate the need to directly address neutrophils as cell-drivers of tissue injury in ARDS.

The low to non-efficacy of dexamethasone in ARDS, despite being an established immune-suppressor that increases monocyte/macrophage efferocytosis (14, 15), reinforces the central role of neutrophils in secondary tissue injury, and in hindsight, can be explained by dexamethasone increasing neutrophil release from the bone marrow and lengthening neutrophil lifespan (16). This therapeutic perspective is supported by efficacy of neutrophil depletion to attenuate acute lung injury (ALI) *in vivo* in preclinical ARDS/ALI models (17, 18), and non-efficacy of monocyte/macrophage depletion which worsened ARDS/ALI in animal models (19). However, as neutrophil-depletion is not clinically feasible and neutrophils exhibit molecular and functional heterogeneity (20), blocking neutrophils to attain efficacy in reducing morbidity or mortality in ARDS has also been elusive (21). Cumulative data suggest that identification of actionable neutrophil-subsets that contribute to feed-forward progression of dysregulated inflammation-induced tissue injury, ie, “rogue” neutrophil subsets, and the target-specific induction of apoptosis in said “rogue” neutrophils are needed. Since neutrophil apoptosis is required for efferocytosis and active initiation of resolution, the targeted induction of apoptosis can eliminate “rogue” neutrophil subsets, initiate efferocytosis and inflammation resolution (22), while sparing non-rogue neutrophil subsets required for defense against infections and/or repair.

Recently, we have identified a targetable “rogue” neutrophil subset expressing the dual endothelin-1/signal peptide receptor (DEspR) on activated CD11b+ neutrophils (23). DEspR+CD11b+ neutrophils were detected in ARDS lung parenchyma, microvasculature and intra-alveolar exudates on post-mortem lung tissue section immunohistochemistry-staining; and peripheral levels correlated with ARDS severity measures, same-day sequential organ failure (SOFA)-scores and day-28 intensive care unit-free days, in contrast to non-correlation of DEspR-negative (DEspR[-]) neutrophil levels (23). *Ex vivo* studies of ARDS patient peripheral neutrophils demonstrated increased survival of DEspR+ neutrophils which was decreased by humanized anti-DEspR antibody incubation (23). *Ex vivo* live cell analysis of rhesus macaque neutrophils confirmed increased survival of DEspR+ neutrophils as observed in ARDS patients, and demonstrated anti-DEspR antibody binding, internalization and induction of apoptosis in DEspR+ neutrophils (23).

Here we test early validation of the DEspR+CD11b+/CD66b+ neutrophil-subset as a potential therapeutic target for ARDS in different LPS-induced acute inflammation/injury models in three species: humans, rhesus macaques, and rats. As Toll-like receptor 4 (TLR4) is the LPS-receptor on neutrophils (24), LPS-induced acute inflammation tissue injury models represent not just endotoxemia, but also represent sterile TLR4-activation by its endogenous ligand, S100A8/A9, an exemplar damage associated molecular pattern (DAMP) increased in acute lung injury which sustains neutrophil activation (25).

To test DEspR+ neutrophils as a potential therapeutic target, we tested 1] whether segmental LPS-challenge in HVs induces DEspR+ neutrophils in bronchoalveolar lavage fluid (BALF), 2] whether LPS-induced transient acute lung injury model in macaques induces DEspR+ neutrophils in peripheral blood and BALF, and 3] whether DEspR-inhibition can reduce LPS-induced DEspR+CD11b+ neutrophil levels and/or hypoxemia. To assess *in vivo* efficacy and safety in a high-mortality acute hyperinflammation state, we tested 4] whether anti-DEspR antibody treatment improves median survival in an LPS-induced encephalopathy model in hypertensive rats, and 5] whether concomitant brain target engagement and bioeffects of anti-DEspR on secondary brain injury support *in vivo* observations.

## Methods

### Anti-DEspR antibodies

We used several blocking anti-DEspR antibodies to demonstrate actionability of the DEspR+ neutrophil subset. For studies in healthy HVs and macaques, we used the recombinant monoclonal humanized anti-DEspR antibody hu6g8 with a hinge-stabilized human-IgG4^S228P^ antibody [Lake Pharma, Inc., now CuriaGlobal.com] which binds to an identical epitope in human, non-human primate, and rat DEspR hormone binding domain (23). For *in vivo* studies in rats, we used the monoclonal anti-rat-specific DEspR murine-IgG1 antibody 10a3. For target engagement and bioeffect studies, we used 10a3 and 6g8, the latter being the monoclonal mouse anti-DEspR antibody precursor of hu6g8 (23). Doses and concentrations used per antibody are listed below per experimental method.

### Healthy human volunteer studies

Three healthy, non-smoking volunteers underwent endobronchial (segmental) LPS challenge and bronchoalveolar lavage. The study was approved by the Ethics Committee of the Hannover Medical School and volunteers gave their written informed consent.

### Segmental LPS challenge and ChipCytometry

The segmental LPS challenge and bronchoalveolar lavage were performed as previously described (26). Briefly, BAL was sampled from the left lower lung lobe at baseline by instilling 5x 20ml of pre-warmed sterile 0.9% saline. Ten ml 0.9% saline and 10 ml LPS solution, 40 ng E.U. resulting in approximately 4 ng/kg body weight LPS in 0.9% saline were instilled into a segment of the lingual lobe (saline) and the right middle lobe (LPS), respectively. After 24 hours (hrs), respective segments were lavaged and cells from the BALF were isolated (26). Cells were applied to ChipCytometry chips (Canopy Biosciences), fixed and stored at 2-8°C until staining.

Fluorescence images were taken at 560 nm and 488 nm before and after staining with CD66b-PE (15 min, 0.4ng/ml, BioLegend) and DEspR-AF488 (hu6g8, overnight, 0.5 ng/ml) at 2-8°C. Analyses were performed using the ZKWApp. Twenty images were segmented automatically followed by manual quality control. Neutrophils were identified based on CD66b positivity. The numbers of DEspR- (approx. < 10.000 AU) and DEspR+ neutrophils (approx. ≥ 10.000 AU) were counted manually. All images were analyzed when, in total, less than 100 neutrophils were found on the images otherwise one representative image was used for analysis.

### Rhesus macaque studies

The studies were custom performed by Envol Biomedical (Primate Products, LLC Protocol Number: VS2001) and conducted according to Primate Products, LLC Standard Operating Procedures and authorized veterinary standards.

### Efficacy of hu6g8 Ab in LPS-induced transient acute lung injury model

Six non-naïve male macaques (~5-7 kg) underwent baseline characterization for physiological measures, flow cytometry and cytokine analysis, followed by random assignment to the following study groups. Group 1 (n = 3): macaques received intravenous (IV) anti-DEspR antibody (hu6g8, 3mg/kg; 30min prior LPS) followed by IV LPS (50 μg/kg). Group 2 mock-treatment control (n = 2): macaques received placebo IV saline followed by IV LPS; Group 3 mock-LPS control (n = 1): macaque received placebo IV saline followed by IV phosphate buffered saline (PBS). Body temperature and O2-saturation by pulse oximetry (SpO2) were measured 0hrs, 4hrs, and 24hrs after LPS challenge. Blood was collected at 0hrs, 2hrs, 4hrs, 8hrs, 24hrs, and 72hrs after LPS challenge for flow cytometry (FCM), and for plasma interleukin 6 (IL-6) and tumor necrosis factor alpha (TNF-α) analyses by ELISA. BALF was collected 4hrs, and 24hrs after LPS challenge. DEspR+CD11b+ neutrophils in whole blood and BALF were detected by flow cytometry following identical protocols as described previously (23). Gating for neutrophils *via* forward scatter/side scatter (FSC[size]/SSC[granularity]) properties was used to distinguish neutrophils from other leukocytes, and to exclude apoptotic neutrophils (smaller FSC or smaller FSC/higher SSC) as previously validated (27, 28).

### Pharmacokinetic analysis of anti-DEspR hu6g8 antibody in macaques

In another macaque study group, pharmacokinetic (PK) analysis of DEspR antibody in macaques (hu6g8, antibody dose 3 mg/kg, n = 2) was done. Human-specific IgG4 levels were assessed by ELISA at 2hrs, 4hrs, 8hrs, 24hrs, 72hrs, 144hrs, 312hrs and 480hrs time points using in-house optimized ELISA. PK data analysis was performed by using PKSolver, an add-in program for pharmacokinetic and pharmacodynamic data analysis in Microsoft Excel (29).

### Live cell imaging of peripheral macaque DEspR+ neutrophils and DEspR[-] leukocytes

Live cell imaging of macaque neutrophils was performed to show AF568-fluorescently labeled anti-DEspR hu6g8 (AF568-hu6g8) antibody binding, internalization, translocation to the nucleus and induction of apoptosis as previously described (23), and to analyze time-course effects on neutrophil adhesion and apoptosis in DEspR+ neutrophils. Briefly, macaque leukocytes were incubated with AF568-hu6g8 antibody, 10 μg/ml, for 20 min in HBSS + 2% FBS on ice to eliminate non-specific endocytosis. Cells were then washed to remove excess antibody, resuspended in RPMI, counted, and seeded onto microfluidic chip channels coated with 2% BSA at 10^8^ cells/ml. Live cell imaging was performed with images obtained every 1-minute across 3 microfluidic chip-chambers in the first 9-hours then every 5-minutes overnight until 24-hours. Standard media change was performed to test for low adhesion (cells wash off) and high-adhesion (cells remain adherent) at 5min and 30min from seeding. Images were visually examined and quantified for cell adhesion and DEspR+/- expression. Apoptotic DEspR+ neutrophils were identified by internalized fluorescent anti-DEspR hu6g8 antibody and cell morphology hallmarks: nuclear/cellular budding. Late apoptosis with secondary necrosis was identified by characteristic condensed nucleus, cell swelling and loss of cell integrity. Loss of cell integrity was defined by entry of impermeant dye, Sytox Green nucleic acid stain (ThermoFisher, Cat. # S7020) which fluoresces only upon binding to DNA. Sytox Green (1:6000 dilution) was added at the 2^nd^ media change at t-30 minutes from seeding of AF568-labeled macaque leukocytes onto microfluidic chips for live-cell imaging.

### Rat studies

Rat studies were performed in accordance with the recommendations in the Guide for the Care and Use of Laboratory Animals of the National Institutes of Health. The protocol was approved by the Committee on the Ethics of Animal Experiments of Boston University School of Medicine (Permit Number: AN-14055) with Category E approval. Euthanasia of study animals was done by removal of vital organs and exsanguination under deep isoflurane anesthesia as stated in the 2013 AVMA Guidelines.

### Survival studies in LPS-induced acute hyperinflammatory encephalopathy rat model

For *in vivo* efficacy studies, we used female Dahl Salt-sensitive hypertensive rats that develop hypertensive spontaneous intracerebral hemorrhage (hsICH) at around 4.5 months of age when exposed to gestational 0.4% NaCl rather than 0.23% NaCl diet in dams, and maintained on 0.4% NaCl regular rat chow (Purina 5001) (30). The first rat in a multi-litter age-matched group to develop acute sICH, the “signal rat”, marked the timepoint to begin the LPS-induced encephalopathy model in hsICH rats. At 19 weeks of age, seventeen anesthetized female hsICH rats received IV bolus-injection of LPS (E coli 0111:B4, Sigma Aldrich, Cat#L2630) at a non-endotoxic LPS-dose of 1.8 mg/kg in saline *via* tail vein followed by immediate injection of anti-DEspR (10a3, 1 mg/kg, n = 8) or saline vehicle control (n = 9) as mock-treatment (mockTx). Rats were monitored until disease progression led to study endpoint.

### *Ex vivo* Study of anti-DEspR effects on survival of *in vivo* LPS-activated neutrophils

After determining optimal timing for blood collection of *in vivo* LPS-activated neutrophils (n = 4), Dahl salt-sensitive hypertensive rats, genetically identical strain for hsICH rat model, (n = 2) were injected IV with 500µl LPS (Sigma Cat#L2630, 1.8 mg/kg in saline) 2hrs prior to blood collection. Five mLs of whole blood were collected in EDTA collection tube (BD Biosciences Cat#366450) under deep anesthesia, followed by euthanasia. The blood was brought to room temperature, layered onto a double gradient Histopaque (Sigma-Aldrich 10771 and 11191 respectively) as per manufacturer’s specifications (Millipore Sigma) and spun 800 x g for 30 minutes. Neutrophils were collected at the 1077/1191 interface, washed with 5 ml RPMI-1640 medium (Sigma Cat# R0883-500 ml) containing 1% BSA. In P96-well plates 50,000 live cells in 200 µL RPMI-1640 containing 1% BSA were incubated with 10a3 and 6g8 monoclonal antibodies (30 µg/ml) for 6 hours at 37°C and 5% CO2 respectively. Each condition was performed with 6 replicates. Live and dead cells were distinguished using trypan blue dye exclusion assay and counted in a phase contrast microscope.

### Study of target engagement and bioeffects

Antibody target engagement and bioeffects were investigated with five hsICH male rats at 12 weeks of age. Group 1 LPS mock-treatment control (n = 2): LPS (IV 1.8 mg/kg *via* tail vein) with no treatment; Group 2 LPS-anti-DEspR treatment (n = 2): with one rat receiving anti-DEspR 10a3 (1.0 mg/kg IV) and the other 6g8 (1.0 mg/kg IV) 7hrs post-LPS injection. Group 3 mock-control (n = 1): saline IV, no LPS. Rat whole brains were collected 24 hrs after LPS injection and weighed after the animals were perfused with 50 ml ice-cold PBS using a large volume syringe *via* the abdominal aorta. Subsequent steps were performed on ice using ice cold buffers. Approximately 2 grams of brain tissue were washed with PBS and minced in a Petri dish containing two volumes of homogenization buffer (20mM Tris-HCl pH 7.4, 250 mM Sucrose, 1 mM EDTA and 1% Protease inhibitor). The tissue suspension was transferred to a 10 ml Wheaton glass homogenizer and homogenized with 10-15 strokes. The tissue lysate was centrifuged at 11,000 x g for 10 minutes at 4°C. The supernatant, containing the post-mitochondrial supernatant protein fraction (PMS fraction), was collected and 500 µl aliquots were frozen at -80^°^C. The remaining pellet (membrane protein fraction) was resuspended in 2 volumes of 0.1M glycine, pH 2.5 until homogeneous. The lysate was centrifuged at 11,000 x g for 10 minutes at 4^°^C. The supernatant, further referred to as the glycine membrane (GM) fraction, was collected containing the eluted murine IgG from the anti-DEspR antibodies infused. Two milliliters of the GM fraction supernatant were transferred to a fresh 15 ml conical tube containing 130 µl of 1M Tris-HCl pH 9.5. The sample was vortexed for 15 seconds before freezing 500 µl aliquots at -80^°^C.

### ELISA for myeloperoxidase, albumin and mouse-specific immunoglobulin

Individual ELISA protocols were performed as per manufacturer’s instructions. Murine IgG anti-DEspR monoclonal antibodies 10a3 and 6g8 were quantified in the brain GM fraction to document brain cell-membrane target engagement using the mouse IgG ELISA kit (LSBio Cat# LS-F10451). Myeloperoxidase (MPO) was quantified in the PMS fraction of the brain tissue as a marker of neutrophil brain infiltrates. Rat albumin was quantified in the PMS fraction as a marker of brain edema. Decrease in MPO and albumin documented target bioeffects respectively. MPO ELISA kit (Hycult Biotech Cat# HK105) and Rat Albumin ELISA kit (AssayPro Cat# ERA2201-1) were used respectively at a 1:10 sample dilution.

### Statistical analysis

Descriptive statistics were obtained and the appropriate statistical tests were applied for the different studies. Kruskal-Wallis with Dunn’s multiple comparison testing was performed to analyze differences in BALF neutrophils among study groups in human volunteers. Paired t-test was used to assess differences in peripheral neutrophil counts and NLR pre- and post-LPS segmental challenge (GraphPad Prism V9.4). For the LPS-ALI macaque study comparing saline mock-treated control vs antibody-treated groups in different parameters across a 24-72-hour time course, we used One Way Repeated Measures ANOVA followed by the Holm-Sidak test for multiple comparisons (SigmaPlot 11.0). For group contingency analysis of neutrophil adhesion and effects of anti-DEspR treatment on DEspR+ macaque neutrophil adhesion, we used Fisher’s Exact test (GraphPad Prism V9.4). For the LPS-encephalopathy rat model survival studies, we used Kaplan-Meier survival curves testing for statistical significance using the Mantel Cox Log Rank Sum test (GraphPad Prism V9.4). For *in vitro* assays of anti-DEspR effects on rat neutrophil survival, and analysis of anti-DEspR antibody target engagement and bioeffects we used One Way ANOVA followed by Holm-Sidak test for multiple comparisons (SigmaPlot 11.0). *P* < 0.05 was considered statistically significant.

## Results

### LPS-induces DEspR+CD66b+ neutrophils in human BALF

**Figure 1:**
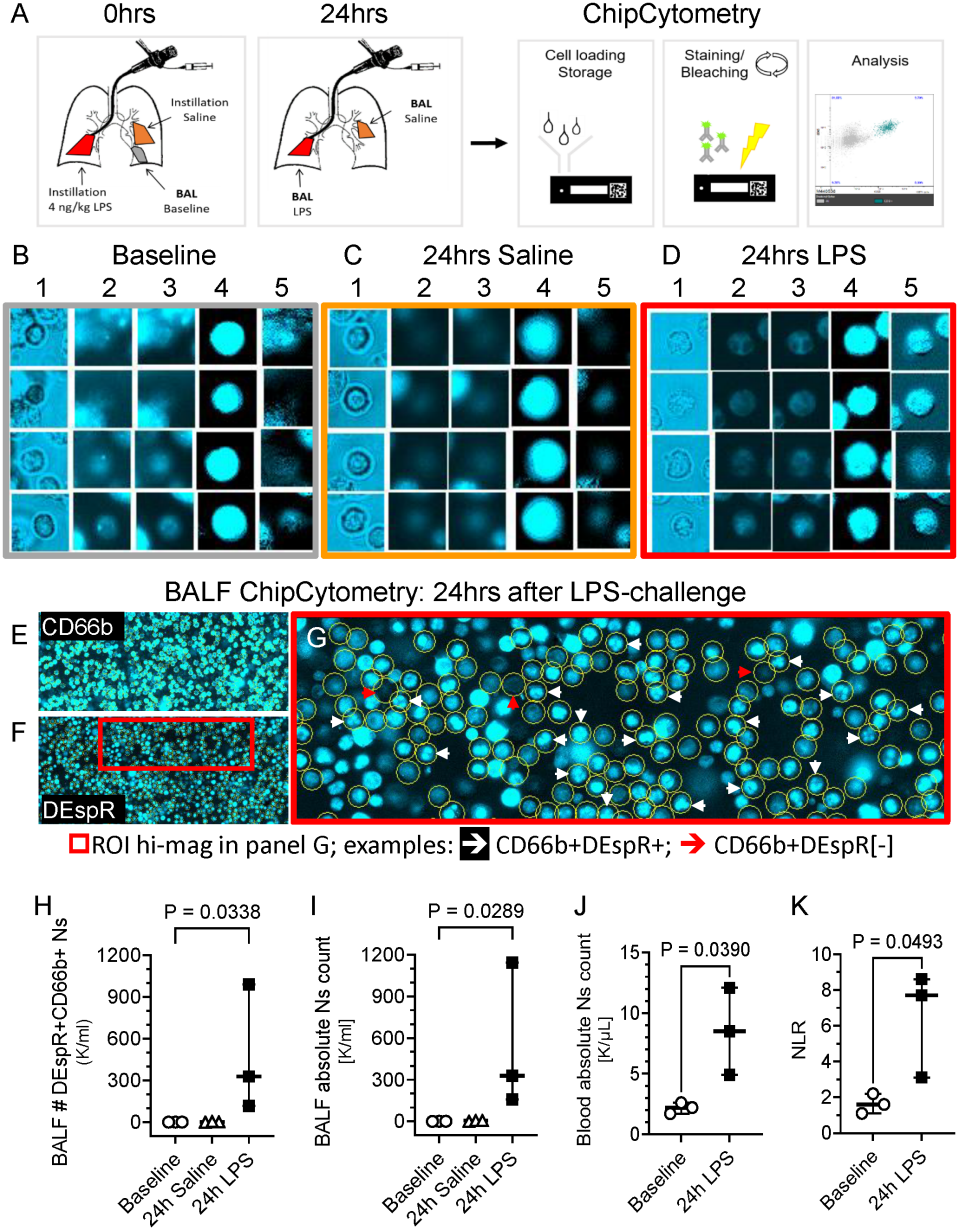
Representative ChipCytometry detection of DEspR+CD66b+ neutrophils. **(A)** Diagram of ChipCytometry analysis in the segmental LPS-challenge model to obtain segment-specific human BALF cells at baseline (left lower lung), 24-hours after saline (right middle lobe) or LPS-challenge (left lingular lobe). **(B-D)** Representative ChipCytometry images from human healthy volunteers at baseline **(B)**, in lung segments with saline instillation **(C)**, and in lung segment with LPS-instillation **(D)**. Columns represent: 1] transmitted light, 2] autofluorescence in phycoerythrin (PE) and 3] Alexa-Fluor (AF)488 channels, 4] CD66b-PE immunofluorescence after subtraction of PE-autofluorescence, 5] DEspR-AF488 immunofluorescence after subtraction of AF488-autofluorescence. Transmitted and fluorescence light images of four representative CD66b+ granulocytes are depicted for each. **(E, F)** Representative low magnification images of ChipCytometry, after subtraction of autofluorescence signals, showing CD66b+ neutrophils **(E)**, and corresponding CD66b+DEspR+ neutrophils, wherein CD66b+ neutrophils are encircled **(F)**. Red boxed region of interest (ROI) shown in panel G in higher magnification. **(G)** Representative image showing CD66b+ neutrophils (encircled in yellow) that are DEspR[-] red (↓) and DEspR+CD66b+ neutrophils with nuclear expression of DEspR white (↓). **(H, I)** Analysis of changes between baseline, 24-hours after segmental-saline and segmental-LPS challenge in BALF **(H)** number (#) of CD66b+DEspR+ neutrophils (Ns) and **(I)** BALF total # of neutrophils (Ns). **(J, K)** Analysis of changes between baseline and 24-hours (24h) post-segmental LPS-challenge in **(J)** peripheral absolute neutrophil (N) counts (K/μL) in HVs and in **(K)** peripheral neutrophil-to-lymphocyte ratio.

To study the DEspR+CD66b+ neutrophil (N)-subset in human BALF, we conducted studies in healthy volunteers. BALF was collected at baseline and 24hrs after segmental challenge with saline or LPS in different lung segments (**Figure 1A**). Using immunofluorescence ChipCytometry, we evaluated CD66b+ and DEspR+ expression on BALF neutrophils in baseline, saline and LPS samples (**Figures 1B-G**). DEspR expression was increased after segmental LPS challenge (mean 86.8% ± SD 10.7%) compared to saline (33.4% ± 20.0%) and baseline (29.8% ± 9.8%) controls. Although DEspR-positive fluorescence intensity is less than CD66b, most CD66b+ neutrophils express DEspR (**Figures 1E-G**). ChipCytometry epifluorescence images also show differential DEspR+ expression patterns in CD66b+ neutrophils: diffuse cell surface and/or cytoplasmic (**Figures 1D**, **1G**) as well as expression in polylobulated neutrophil nuclei (**Figure 1G**).

Quantitation detected significant increase in number of DEspR+CD66b+ neutrophils (**Figure 1H**) as well as in total neutrophil counts (**Figure 1I**) in BALF after segmental LPS challenge compared to baseline levels (*P* < 0.05, respectively). We also detected CD66b[-]DEspR^BRIGHT^ cells with larger diameters than CD66b+ neutrophils suggestive of alveolar macrophages in the BALF sample. As expected, segmental LPS-challenge also significantly increased peripheral absolute neutrophil counts (**Figure 1J**) and neutrophil lymphocyte ratio (NLR, **Figure 1K**) between pre-LPS and post-LPS challenge samples, paired t-test *P* < 0.05 respectively.

### Role of DEspR+CD11b+ neutrophil-subset in LPS-ALI model in rhesus macaques

**Figure 2:**
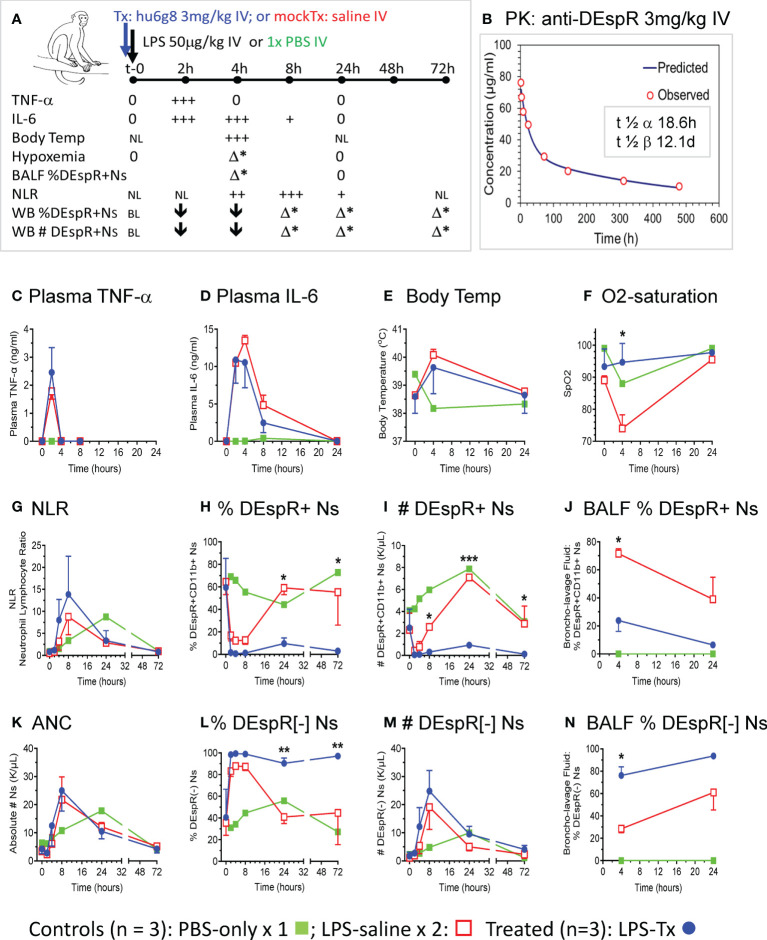
*In vivo* analysis in LPS-transient ALI rhesus macaque model studies. **(A)** Diagram of *in vivo* study along timeline, t-0 (baseline) to 72hrs with measures obtained. LPS [50 μg/kg] was infused intravenously (IV) after a IV single dose of humanized anti-DEspR (hu6g8) antibody (n = 3), or mock-treatment (Tx) saline for placebo LPS-control (n = 2). Non-LPS PBS-only (n = 1) served as negative control. Measures obtained at designated time points: plasma TNF-α, IL-6; body temperature (temp), hypoxemia (% O2-saturation), flow cytometry analysis of % DEspR+CD11b+ neutrophils (Ns) in BALF, bronchoalveolar lavage fluid and whole blood samples, and absolute total number (#) of peripheral DEspR+CD11b+ neutrophils (K/μL) in whole blood samples. BL, baseline levels, NL, normal levels; 0, zero levels; ↓, decrease; + to +++, increased levels with higher +++; h, hrs; Δ*, significant change between treated and saline mock-treated macaques in corresponding parameter. **(B)** Pharmacokinetic analysis of anti-DEspR hu6g8 in macaques (n = 2) given 3 mg/kg dose IV. Half-life of distribution, t ½ α: 18.6 hours (h), half-life of elimination, t ½ β: 12.1 days **(D)**. Time points for human IgG4 ELISA measurements in another study group of macaques (n = 2): 2-, 4-, 8-, 24-, 72-, 144-, 312-, 480-hours. Time course of averages of different measures per study group plotted **(C-N),** n = 3 LPS + treatment (LPS-Tx), n = 2 LPS-saline control, n = 1 no-LPS, no treatment control. One-way repeated measures ANOVA, with Holm-Sidak pairwise comparisons performed, significant *P* values notated respectively. **(C)** ELISA plasma TNF-α (ng/ml); **(D)** ELISA plasma interleukin (IL)-6 (ng/ml); **(E)** body temperature (°C); **(F)** O2 saturation (SpO2): *, *P* = 0.03. **(G)** neutrophil-lymphocyte ratio (NLR), **(H)** peripheral % DEspR+CD11b+ neutrophils (Ns): 24h (*) *P* = 0.02; 72h (**) *P* = 0.03); **(I)** peripheral total number (#) of DEspR+CD11b+ Ns (K/μL): 8h (*) *P* = 0.03; 24h (***) *P* = 0.0001; 72h (*) *P* = 0.01. **(J)** BALF % DEspR+CD11b+ Ns: 4h: (*) *P* = 0.02. **(K)** Peripheral absolute neutrophil counts (ANC), **(L)** % DEspR-negative [-] neutrophils (Ns): 24h (**), *P* = 0.008, and 72h (**) *P* = 0.007. **(M)** Peripheral number (#) of DEspR[-] Ns, and **(N)** BALF % DEspR[-] Ns: 4h (*) *P* = 0.02.

Using the LPS-induced transient ALI macaque model, we compared anti-DEspR hu6g8 pre-treated macaques (n = 3, blue lines) *vs* saline pre-treated placebo (n = 2, red lines) and PBS-only treated (n = 1, green line) macaques (**Figure 2**). From t-0 baseline to 72hrs, we monitored multiple parameters to document the effects of LPS on key cytokines TNF-α and IL-6, key pathophysiological measures body temperature and O2-saturation, as wells as neutrophil measures of DEspR+CD11b+ neutrophils in the circulation and in BALF (**Figure 2A**). Plasma levels of anti-DEspR hu6g8 were monitored in two additional animals resulting in clinically feasible pharmacokinetics with half-life of distribution of 18.6 hours, and half-life of elimination of 12.1 days (**Figure 2B**).

To document equivalent LPS-induced acute inflammation, we measured cytokine response in test and control macaques, confirming equivalent LPS-challenge in both anti-DEspR hu6g8 treated (blue) and saline placebo control macaques (red, **Figures 2C, D**). Increased plasma TNF-α levels peaked at 2hrs dropping back to baseline levels by 4hrs in both control and treated macaques (**Figure 2C**). IL-6 levels peaked between 2-4hrs in both control and treated animal groups (**Figure 2D**), decreased by 8hrs, and dropped back to baseline at 24hrs consistent with a transient ALI model. As expected, development of fever by 4hrs coincided with peak IL-6 levels (**Figure 2E**).

Moderate to severe hypoxemia (SpO2 < 80%), a hallmark of ALI indicating secondary tissue injury, was observed in saline mock-treated control macaques after 4hrs, in contrast to significant reduction in anti-DEspR hu6g8 treated macaques (*P* = 0.03) (**Figure 2F**). We note the resolution of hypoxemia by 24hrs in both groups concordant with transient ALI modeling (**Figure 2F**). PBS-only control showed a decrease in O2-saturation to 90%, however, this can be attributed to effects of anesthesia during bronchoscopy at 4hrs for BALF collection.

Corresponding analysis of peripheral neutrophil-lymphocyte ratio (NLR) showed elevation of NLR by 4hrs peaking at 8hrs, decreasing by 24hrs and dropping back to baseline at 72hrs (**Figure 2G**). No differences were observed between anti-DEspR treated and non-treated macaques in peripheral NLR, as well as in absolute lymphocyte and absolute monocyte counts respectively in this LPS-transient ALI model. In contrast, flow cytometric analysis of % and absolute number (#) of DEspR+CD11b+ neutrophils in whole blood and BALF showed significant differences (Figure H-J). Analysis of whole blood showed peak decrease in % DEspR+CD11b+ and # DEspR+CD11b+ neutrophils at 2hrs and 4hrs (**Figures 2H, I**). Increased % DEspR+CD11b+ neutrophils was observed in BALF at 4hrs compared to 24hrs (**Figure 2J**). We note that BALF neutrophil count and hypoxemia were checked only at 4hrs and 24hrs in compliance with approved institutional protocol for humane use of animals (macaques). Notably, analysis of peripheral blood DEspR+CD11b+ neutrophil profiles (**Figures 2H, I**) showed a decrease at 2-4hrs after intravenous LPS challenge in both saline mock-treated and hu6g8-treated macaques. The decrease in peripheral DEspR+ neutrophils coincides with an increase in % DEspR+CD11b+ neutrophils in BALF at 4hrs compared to PBS-only control (**Figure 2J**), altogether suggesting the influx of peripheral pulmonary marginated neutrophils into the lung BALF upon systemic LPS-induced acute inflammation.

Comparative analysis shows that anti-DEspR hu6g8 treatment significantly decreased the % of DEspR+CD11b+ neutrophils in BALF at 4hrs compared with saline mock-treated controls (*P* = 0.02, One Way RM ANOVA) (**Figure 2J**), which coincides with prevention of hypoxemia in hu6g8-treated macaques (**Figure 2F**). In the circulation, flow cytometry detects low levels of non-apoptotic DEspR+CD11b+ neutrophils at 8h and 72hrs whether measured as % or absolute number of DEspR+CD11b+ neutrophils (**Figures 2H, I**). As hu6g8 is internalized upon binding to DEspR+ neutrophils subsequently inducing neutrophil apoptosis (23), observed decreased levels of DEspR+CD11b+ neutrophils represent low levels of *de novo* or remaining non-apoptotic DEspR+ neutrophils.

Analysis of absolute total number of circulating neutrophils (**Figure 2K**) and DEspR[-] CD11b+ neutrophils in the circulation as % of total neutrophils (**Figure 2L**) and number of DEspR[-]CD11b+ neutrophils (**Figure 2M**) show no decrease with anti-DEspR hu6g8 treatment. This is concordant with the specificity of anti-DEspR antibodies (31) and a projected mode-of-action for target-specific effects and safety through sparing of DEspR[-] CD11b+ neutrophils activated in response to pathogens.

### Analysis of adhesion profile of macaque DEspR+ neutrophils during live cell imaging

**Figure 3:**
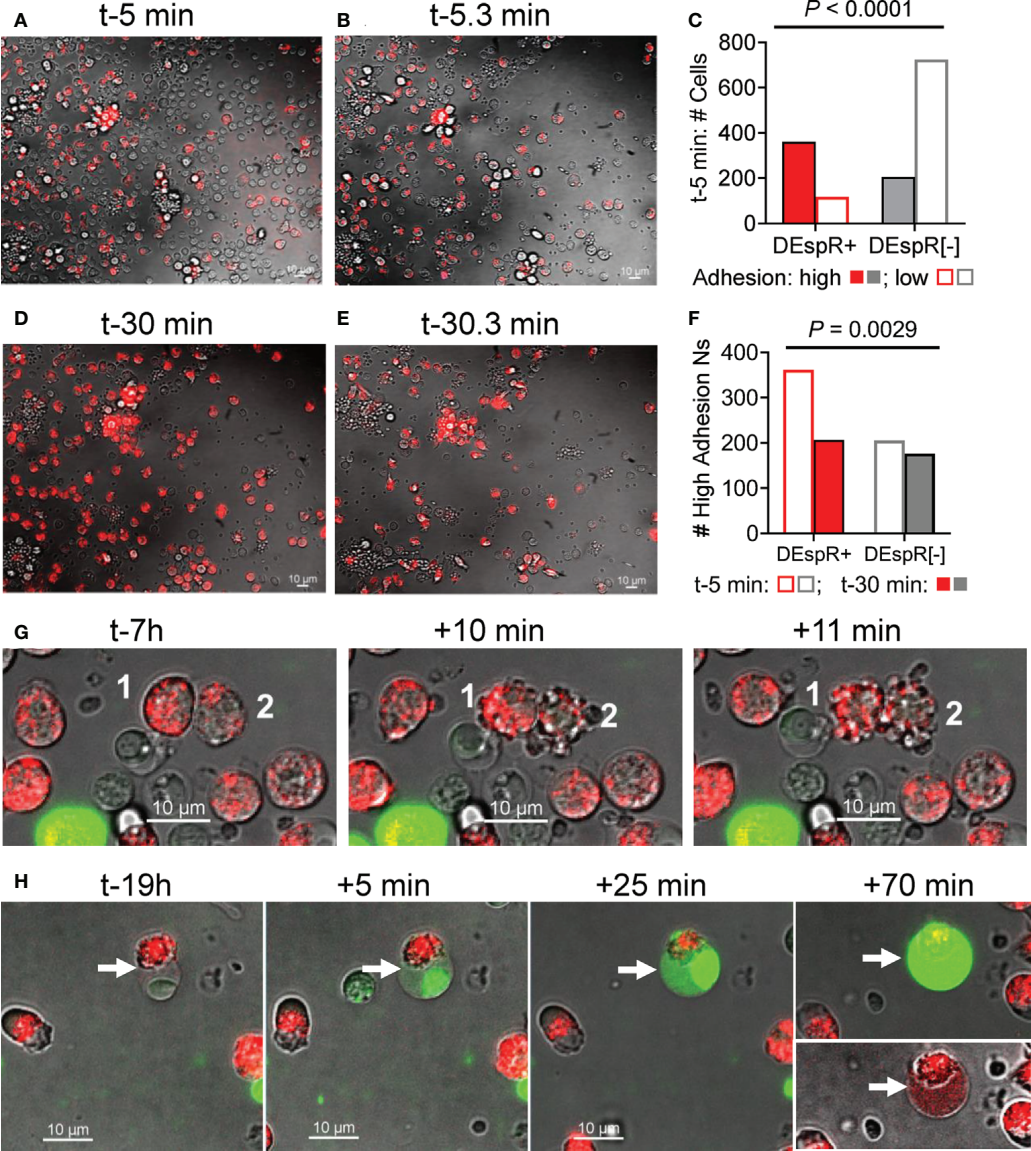
Analysis of neutrophil adhesion during live cell imaging. **(A)** Representative live-cell imaging snapshot showing DEspR+ neutrophils (with bound AF568-hu6g8) and unbound DEspR[-] neutrophils/leukocytes adhered onto the microfluidic-chip channels at t-5 min from seeding, before media change. **(B)** Representative live-cell imaging snapshot immediately after media change t-5.3 min detecting high-adhesion leukocytes (adhesion resists media change) and concomitant loss of leukocytes (low adhesion). **(C)** Graph showing that majority of high-adhesion neutrophils (solid bars) are DEspR+ (red); majority of low adhesion cells (open bars) are DEspR[-] (grey), *P* < 0.0001, Fisher’s Exact test. **(D)** Representative live-cell imaging snapshot at t-30 min with internalized AF568-anti-DEspR hu6g8 moving towards focal z-plane preset at mid-nuclear level during live-cell imaging. **(E)** Representative snap shot of high-adhesion cells after media change among DEspR+ and DEspR [-] neutrophils/leukocytes, and concomitant loss of low-adhesion cells. **(F)** Bar graph showing loss of DEspR+ neutrophils at ~t-30.3 min from seeding and after media change. Fisher’s exact test *P* value = 0.0029. Bar = 10 μm. **(G)** Representative serial live cell imaging snapshots at t-7h showing DEspR+ neutrophils fluorescing with internalized AF568-anti-DEspR hu6g8 and exhibiting nuclear and cellular budding characteristic of apoptosis within 10min from start of cell budding. **(H)** Representative serial live cell imaging snapshots at t-19h showing late apoptosis characteristics with nuclear condensation, and increasing cell swelling and permeability marked by increased staining with impermeant Sytox Green DNA-stain over 70 minutes.

To study adhesion functionality of DEspR+ neutrophils as a potential mode-of-action for LPS-induced increased influx of DEspR+ neutrophils into BALF, we analyzed neutrophil “intrinsic adhesion to hard-surfaces” (32) of DEspR+ and DEspR[-] macaque peripheral blood leukocytes seeded onto microfluidic chips, which were pre-incubated with AF568-labeled anti-DEspR hu6g8 antibody. Using live-cell imaging, adhesion of macaque neutrophils to the chip matrix “hard-surface” was assessed before and after media change. Low adhesive cells were washed off while high adhesive cells remained on the chip. At time-5 minutes (t-5min) from seeding, majority of DEspR+ neutrophils exhibited high-adhesion properties in contrast to DEspR[-] neutrophils and other leukocytes, the latter being the majority low-adhesion cells which were lost upon media change (**Figures 3A, B**). Differences by contingency group analysis were significant *P* < 0.0001 (**Figure 3C**). However, at t-30 min from seeding, the reverse was observed after media change: high-adhesion DEspR+ neutrophils were lost more than high-adhesion DEspR[-] cells, *P* = 0.0029 (**Figures 3D–F**). The greater fluorescence-intensity in DEspR+ neutrophils at t-30min (**Figure 3D**) compared to t-5min (**Figure 3A**) indicates the movement of internalized AF568-DEspR towards the focal plane of live cell imaging (pre-set at mid-nuclear z-plane). The loss in high-adhesion property of DEspR+ neutrophils at t-30min from seeding in contrast to t-5min suggests a progressive decline in adhesion with approximately 2x-longer duration of DEspR inhibition at t-30min.

### Analysis of anti-DEspR induced apoptosis in macaque DEspR+ neutrophils ex vivo

To corroborate induction of apoptosis in DEspR+ neutrophils by anti-DEspR hu6g8 during live cell imaging as previously reported in macaque peripheral neutrophils (23) and pancreatic tumor cells (31), we assessed the time course of classical apoptosis-associated cellular changes in anti-DEspR hu6g8-treated macaque peripheral blood neutrophils by live cell imaging *ex vivo* over 24-hours. We observed non-synchronous internalization and translocation to the z-plane of the nucleus of the antibody over time, as well as a non-synchronous progression to apoptosis-associated cellular and nuclear blebbing/budding (**Figure 3G**). With increased time, we observed increased number of DEspR+ neutrophils exhibiting cellular features of late apoptosis: condensed nucleus, cell swelling and intensified cell-impermeant SytoxGreen staining (**Figure 3H**). Sytox Green staining confirms apoptosis-associated highly condensed nuclei, and surprisingly, shows DNA dispersed in the cytoplasm in late apoptosis. We also note that on live-cell imaging, DEspR+ neutrophils exhibiting late apoptosis changes (**Figure 3H**) did not go through the cellular-nuclear blebbing/budding phase (**Figure 3G**) suggesting separate apoptosis-associated phenomena.

### Efficacy and safety of DEspR inhibition in high mortality LPS-encephalopathy model

**Figure 4:**
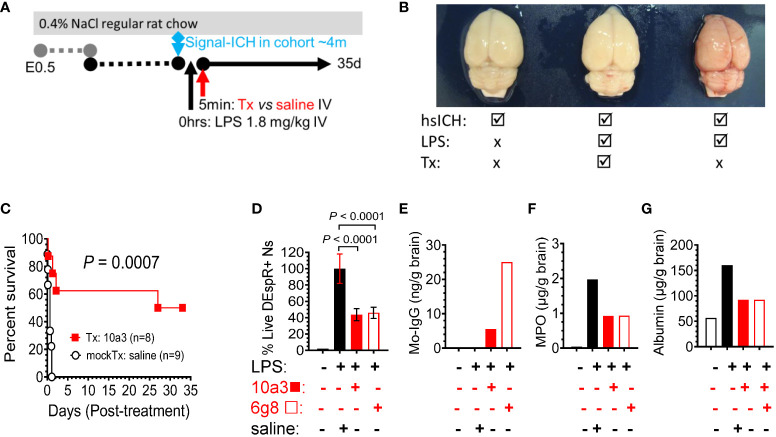
DEspR+ neutrophil-subset roles in LPS-induced tissue injury model. **(A)** Diagram of LPS-induced multi-organ encephalopathy rat model in hsICH-prone rats. Regular salt-challenge (0.4% NaCl regular rat chow) from embryonic day 0.5 (E0.5) in Dahl Salt-sensitive hypertensive rat inducing hypertension-associated neutrophil/endothelial activation. Sub-endotoxic dose of LPS (1.8 mg/kg IV) was infused in study rats after observation of the 1^st^ intracerebral hemorrhage event in the age-matched rat cohort around 4m of age (signal ICH ~4m). After LPS was infused, treatment (Tx), either anti-DEspR mAb 10a3 or 6g8 as notated, or mock-treatment (mockTx) saline (vehicle) was given. Treated rats with full recovery were monitored until 35 days (35d). **(B)** Representative post-mortem images of rat brains after intravascular blood volume replaced with 1X PBS. Left, non-LPS-challenged brain; Middle: anti-DEspR (10a3) mAb treated brain after LPS-infusion. Right: LPS-challenge mock-treated control. **(C)** Kaplan-Meier survival curve of treated (Tx: 10a3, 1 mg/kg IV, n = 8) vs saline mock-treated (mockTx, n = 9) LPS-challenged hsICH rats. Log Rank Mantel-Cox test: *P* = 0.0007; hazard ratio (Mantel-Haenszel 10.2, 95% CI of ratio: 2.67 – 39.25). **(D-G)** Analysis of anti-DEspR mAb target engagement and bioeffects. Study groups are designated (+ or -) per agent (left to right): black open bar = control no LPS and no treatments, black bar = reference control LPS + saline, red bar = LPS + 10a3; red open bar = LPS + 6g8 **(D)** Minimal to no DEspR+ neutrophils in no-LPS rat control. Compared to LPS + saline control (100% reference), anti-DEspR mAbs 10a3 and 6g8 reduce survival of DEspR+ neutrophils (n = 6 replicates/group), concordant with target engagement and bioeffects in peripheral DEspR+ neutrophils *ex vivo*. **(E)** Brain membrane-bound protein levels of mouse-specific immunoglobulin (IgG) in 10a3 or 6g8 mAbs showing brain target engagement, 6g8 > 10a3. **(F, G)** Analyses of brain non-membrane bound proteins by ELISA detect reduced brain levels of **(F)** neutrophil-derived myeloperoxidase (MPO) in 10a3 and 6g8-treated rat brains compared to LPS + saline mockTx rat brain, and **(G)** reduced levels of albumin as a marker of edema in the brain. ng/g brain, nanogram per gram brain; μg/g brain, microgram per gram brain.

To robustly test the efficacy and safety of DEspR inhibition in neutrophil-mediated secondary tissue injury, we used the LPS-encephalopathy rat model with high-mortality phenotype in order to be able to use survival as primary endpoint of efficacy (**Figure 4A**). We prioritized this endpoint as it is the critical endpoint required for clinical trial-based approvals. Analysis of post-mortem brains corroborates the LPS-induced hemorrhagic encephalopathy phenotype in the model comparing no LPS hsICH-control, LPS-anti-DEspR treated, and LPS-saline (vehicle) mock-treated rat brain (**Figure 4B**). Kaplan-Meier Survival curve analysis shows that anti-DEspR 10a3 antibody treatment given shortly after LPS-intravenous infusion significantly increased median survival (Kaplan-Meier *P* = 0.0007) attaining full recovery in 50% of the treated LPS-encephalopathy model, in contrast to mock-treated vehicle (saline) controls (**Figure 4C**). Efficacy in increasing median survival indicates concomitant safety.

To determine bioeffects on DEspR+ neutrophils, we demonstrate that both anti-DEspR antibodies, 10a3 and 6g8, decreased LPS-activated rat neutrophil survival *ex vivo* (*P* < 0.0001, One way ANOVA followed by multiple comparisons test, **Figure 4D**). To assess target engagement and bioeffects of *in vivo* DEspR-inhibition, we analyzed treated LPS-encephalopathy rat brain membrane-bound proteins and detected mouse-specific IgG indicating target engagement, in contrast to no anti-DEspR murine IgG in non-treated control rat brain (**Figure 4E**). Testing for bioeffects 7hrs after infusion, we analyzed brain non-membraned bound proteins by ELISA and detected decreased brain MPO levels to 50% of vehicle-treated control at 7hrs indicating decreased brain neutrophil infiltrates (**Figure 4F**), and decreased brain albumin levels indicating decreased brain edema (**Figure 4G**). Together with *in vivo* efficacy in increasing median survival, ELISA data support the expected *in vivo* target engagement and bioeffects of anti-DEspR antibody in the LPS-encephalopathy rat model.

## Discussion

The consistent detection of the DEspR+ neutrophil-subset across three species in different LPS-induced neutrophilic inflammation models with increasing severity – 1) segmental neutrophilic inflammation and pulmonary-vascular barrier disruption model in human volunteers (33), 2) transient hypoxemia/ALI in macaques, and 3) high-mortality encephalopathy in rats – independently corroborate the neutrophil-subset with surface immunotype DEspR+CD11b+/CD66b+. These results confirm identity of this subset as detected in patients with ARDS, COVID-19-ARDS (23), and more recently, in spontaneous intracerebral hemorrhage (sICH) patients (34). Detection of DEspR+CD66b+ neutrophils, with differential cellular localization in healthy volunteer BALF 24h after segmental-LPS challenge is concordant with previous studies showing a] DEspR+CD11b+MPO+ neutrophils in intra-alveolar exudates with cell membrane, cytoplasmic and nuclear expression in postmortem lung tissue sections from ARDS patients (23), and DEspR+ tumor cells (31). Live cell imaging studies of anti-DEspR antibody binding, internalization and nuclear translocation support the notion that DEspR undergoes membrane-to-nuclear shuttling, which is availed of as mode-of-action for the antibody.

The demonstrations of efficacy of DEspR inhibition *in vivo* in two independent animal LPS-inflammatory models in reducing neutrophil-induced secondary tissue injury damage, 1) hypoxemia in macaques and 2) brain edema and encephalopathy in hypertensive rats, together indicate a pathophysiological role of the DEspR+ rogue neutrophil subset. More specifically, results support the hypothesis that LPS-induced DEspR+CD11b+ neutrophils contribute to the disruption of blood-tissue barriers, and provide experimental support for the strong correlation (Rs > 0.7) of DEspR+ “rogue” neutrophil-subset with SOFA-score in ARDS (23) and with 90-day modified Rankin Scale score for outcome in sICH patients (34).

As efficacy of DEspR-inhibition was observed without adverse effects in the presence of acute inflammation for both humanized and precursor murine anti-DEspR antibodies, data support the potential clinical feasibility of DEspR+ subset-specific inhibition. As efficacy was observed in the acute setting requiring quick-response in 24-hour lethal LPS-encephalopathy rat model, albeit to 50%, data support the potential clinical relevance of DEspR+ subset-specific inhibition. Equally important, the observed subset-specific sparing of DEspR[-] neutrophils provides a mode-of-action for inherent safety: while DEspR+CD11b+ ‘rogue’ neutrophils are blocked, DEspR[-]CD11b+ neutrophils are ‘spared’ to carry on homeostatic neutrophil-mediated surveillance and defense mechanisms. These observations are concordant with prior observations that low DEspR+ neutrophil counts in ARDS (23) and ICH (34) patients are associated with recovery respectively, and that healthy donor neutrophils are DEspR[-] (23).

Since anti-DEspR hu6g8 has been shown to bind, internalize, translocate to the nucleus over time, and induce apoptosis in macaque DEspR+ neutrophils as observed in prior *ex vivo* studies (23), as well as in pancreatic cancer cells (31), *in vivo* efficacy of anti-DEspR in transient LPS-ALI and high-mortality LPS-encephalopathy models in this study provide further proof-of-concept data that induction of neutrophil apoptosis is the therapeutic path to attaining neutrophil function shutdown, efferocytosis and active resolution of inflammation (35–37). The therapeutic targeting of neutrophils as drivers of secondary tissue injury is distinguished from the inhibition of different pathophysiological downstream events – including endothelial and epithelial injury – which have failed in clinical trials (4–13).

Convergence of observations with other candidate therapies showing improvement in preclinical models of hyperinflammation LPS-ALI or bleomycin-ALI models *via* induction of neutrophil apoptosis, such as roscovitine (CDK2, 7, 9 inhibitor) (38)or AT7519 (39), and necrolysin (Nec-1, inhibitor of necroptosis) (40), provide further support for neutrophil-targeted therapeutic approaches. Interestingly, all three – anti-DEspR (31), roscovitine (38) and necrolysin (40) downregulate pro-survival Mcl1 required for neutrophil extended survival (41), a concordance that corroborates observed preclinical efficacies respectively. However, limitations from broad, non-subset-specific, neutrophil effects may bring challenges as roscovitine also inhibits neutrophil progenitors (42), and as necrolysin also induces apoptosis in the liver causing liver injury and reduced survival in a sepsis-rat model (43). Given neutrophil functional and molecular heterogeneity in inflammation (37), anti-DEspR therapy, as a subset-specific targeting antibody, can open the door to a potential pathway to the clinic as monotherapy or as partner in combinatorial neutrophil-targeting therapies. More studies are needed to test these hypotheses.

### Limitations of the study

There are limitations in this study. We recognize the small group numbers in segmental LPS-challenge model in human HVs, however, the data are unequivocal for presence/absence of DEspR+ neutrophils in BALF. We recognize the small group number in the LPS-transient ALI model in rhesus macaques, however the data show significant DEspR inhibition effects by repeated measures ANOVA and multiple comparisons statistical testing. Due to stress minimization institutional protocol stipulations, more time points for BALF collection under anesthesia for flow cytometry analysis, and more frequent monitoring of hypoxemia in the LPS-ALI macaque study were not done. In the LPS-hsICH rat model, treatment was given shortly after LPS-challenge and survival analysis was performed without blood sampling so as to avoid stress-confounders from isoflurane anesthesia exposure imperative in survival studies. Due to limited samples and time constraints to ascertain robust neutrophil analyses, we were unable to dissect other mechanisms involved, NET-forming neutrophils, endothelial-neutrophil adhesion and interactions, and DEspR+CD11b+ monocytes.

## Conclusion

This pilot study confirms the DEspR+CD11b+/CD66b+ neutrophil subset, provides insight into neutrophil subset-specific roles, and validates neutrophil subset-specific inhibition as a clinically feasible therapeutic approach. This pilot study provides basis to further study the targeted inhibition of DEspR+CD11b+ neutrophils as novel therapy for neutrophil-mediated acute tissue injury, as well as to identify and test other neutrophil subset-specific targeting therapies and combinations of neutrophil-targeting therapies.

## Data availability statement

The raw data supporting the conclusions of this article will be made available by the authors, without undue reservation.

## Ethics statement

The studies involving human participants were reviewed and approved by the Ethics Committee of the Hannover Medical School and volunteers gave their written informed consent. The patients/participants provided their written informed consent to participate in this study. The Rhesus monkey studies were approved by the Committee on the Ethics of Animal Experiments at Envol Biomedical, and custom performed by Envol Biomedical (Primate Products, LLC Protocol Number: VS2001) and conducted according to Primate Products, LLC Standard Operating Procedures and authorized veterinary standards. Rat studies were performed in accordance with the recommendations in the Guide for the Care and Use of Laboratory Animals of the National Institutes of Health. The protocol was approved by the Committee on the Ethics of Animal Experiments of Boston University School of Medicine (Permit Number: AN-14055) with Category E approval.

## Author contributions

Design and overall supervision of study: SC, MM, JH, VH, NR-O; study of BALF samples from human healthy volunteers: SC, JH, MM; analysis of data on macaque studies: NR-O, VH; rat LPS-model studies: GT, KP, NR-O, VH; manuscript writing: SC, MM, JH, VH, NR-O; manuscript review: all authors. All authors contributed to the article and approved the submitted version.

## Funding

Funding for this study has been provided by: a] Fraunhofer ITEM grant for segmental LPS-challenge in human healthy volunteers, b] NControl therapeutics for rhesus macaque studies performed at ENVOL Biomedical; c] Ellison Foundation, Boston MA, to VH for LPS-induced multi-organ failure/encephalopathy rat model studies, d] Boston Biomedical Innovation Center Drive Grant NIH NHLBI 5U54HL 119145-07. All funding agencies did not participate in the studies, data analyses or manuscript preparation.

## Acknowledgments

We acknowledge the primate study services of Envol Biomedical [www.envolbio.com/primate], for the LPS-induced transient ALI model and PK-studies in rhesus macaques.

## Conflict of interest

Boston University holds awarded and pending patents on DEspR. VH and NR-O are co-inventors filed by Boston University. These patents comprise the option granted for exclusive license to NControl Therapeutics, Inc. VH, NR-O are scientific co-founders of NControl Therapeutics, and paid consultants with equity in NControl Therapeutics, Inc. NControl Therapeutics was not involved in the design, conception, data interpretation, or manuscript preparation.

The remaining authors declare that the research was conducted in the absence of any commercial or financial relationships that could be construed as a potential conflict of interest.

## Publisher’s note

All claims expressed in this article are solely those of the authors and do not necessarily represent those of their affiliated organizations, or those of the publisher, the editors and the reviewers. Any product that may be evaluated in this article, or claim that may be made by its manufacturer, is not guaranteed or endorsed by the publisher.
